# Editorial: Preterm follow-up: the progression of neonatal care

**DOI:** 10.3389/fped.2023.1265235

**Published:** 2023-09-19

**Authors:** J. B. Muller, S. Marret

**Affiliations:** ^1^ Department of Neonatal Medicine, University Hospital of Nantes, Nantes, France; ^2^ LIFT cohort 44000 Nantes, 4 Loire Infant Follow-up Team (LIFT) Network, Pays de La Loire, Nantes, France; ^3^ Department of Neonatal Paediatrics and Intensive Care and Neuropaediatrics, Rouen University Hospital, Rouen, France; ^4^ INSERM, Rouen University Institut National de la Santé et de la Recherche Médicale Unité, Rouen, France; ^5^ Genomic and Personalized Medicine in Cancer and Neurological Disorders, Institute for Research and Innovation in Biomedicine (IRIB), Normandie University, Rouen, France

**Keywords:** preterm, follow-up, long term, developmental care, progression of care

**Editorial on the Research Topic** Preterm follow-up: the progression of neonatal care

To what extent has the evolution of neonatal care impacted long-term outcomes of preterm infants? In the last few decades, the prevalence of severe cerebral palsy (CP) in very preterm children has slowly decreased with advancements in perinatal care (1). However, the overall prevalence of CP and developpemental coordination disorders remain consistent (2). Nearly half of all very and moderate preterm children of the EPIPAGE 2 cohort followed up at five-years has neurobehavioral developmental disorders (NDD) including developmental coordination skills, neurovisual functions, cognitive performance and/or behavioral regulation difficulties (1) Accumulating data suggest an additional influence of psycho-social, epigenetic and/or cellular factors in the occurrence of NDD in preterm newborns (3).

Burden of care, use of neonatal drugs and nosocomial infections in neonatal intensive care unit (NICU) may directly impact on long-term outcomes. The typical management of RDS involved mechanical ventilation that may contribute to the development of chronic lung disease. The progression of non-invasive ventilation support, the rational use of intratracheal administration of surfactant and sedative drugs limit respiratory side-effects and may improve long-term outcome. In this topic “The long-term outcomes of preterm infants receiving non-invasive high frequency oscillatory ventilation” (Li et al.), identified similar respiratory effects compared receiving nasal intermittent postive pressure ventilation and nasal continuous positive airway pressure. Advances in neonatal care are also applied to breasfeeding support and nutritional practices. Beginning and achieving full enteral nutrition in a shorter time frame limits the use of central lines, reduces nosocomial infection rates and neonatal morbidity that may impact long-term outcomes. For instance, the association between nosocomial infections and behavioral difficulties in childhood is discussed. No association is described in EPIPAGE 2 cohort whereas others demonstrate the opposite, such as in the study published in this topic “Neonatal sepsis is associated with behavioral abnormalities in very low birthweight infants at preschool age” (Giordano et al.).

In addition to technical advances, the role of NICU environment in affecting stress, behaviour and maturation of preterm newborns is crucial. Developmental care based on family centered care are designed to minimize the stress during NICU stay but also to support neurodevelopment of preterm infants. Benefits on short term outcome such as chronic lung disease or length stay are highlighted, but the Impact on long-term neurodevelopment of developmental care program needs to be clarified. Peters KL et al. (Edmonton NIDCAP Trial) show an impact of NIDCAP on 18-month neurodevelopmental outcomes (4), whereas Ohlsson and Jacobs, in a meta analysis do not find any impact on neurodevelopmental of pretem at 18 months (5). New precision-medicine studies based on effect on parenting on long term outcome is required. In this way, you will find in this topic the CALIN protocol “Protocol for a prospective multicenter longitudinal randomized controlled trial (CALIN) of sensory-tonic stimulation to foster parent child interactions and social cognition in very premature infants” (Guittard et al.).

Neonatal assessment of brain structure and architecture with conventional imaging techniques is inaccurate to predict long-term NDD. New imaging techniques may be challenging to explore the atypical neurodevelopmental trajectory of the preterm infant, as discussed in this topic in the manuscript of Gire et al. “A correlation between Magnetic Resonance Spectroscopy (1-H MRS) and the neurodevelopment of two-year-olds born preterm in an EPIRMEX cohort study”).

Systematic follow-up assessment is of a great concern and must also evolve. Standardized and validated scales in assessment of cerebral palsy (General Movements Asessment, Amiel-Tison Neurological Assessment), developmental coordination disorder (Movement Assessment Battery for Children, MABC-2), sensory disabilities (visual acuity, hearing threshold) and cognitive impairment (Wechsler scale) remain difficult to implement despite clinical practice guidelines. Furthermore, executive functions and behavioral regulation are especially affected in preterm children. Executive functions are a set of mental skills that include working memory, flexible thinking, and self-control more recently defined in a theorical model by Diamond (6). Few tools are available to assess impact of executive functions disorder on the daily life of the child. In this topic “Assessing of executive functions in daily life in preterm children in 3–4 year-old from the “Behavior Rating Inventory of Executive Function-Preschool version (BRIEF-P) questionnaire”” (Reynold et al.) is a study on the assessment of executive challenges. Indeed, child daily life of ex-preterm remains an area of concern, in particular quality of life. Parental education and involvement in follow-up and parents' views on the child's daily life are an essential component of care and of future research, here you will find “Neurodevelopment at seven years and parents' feelings of prematurely born children” (Mercier et al.).

Preterm birth remains a health priority as it is the primary cause of infant death in low-, middle- and high incomes countries. It is one of the main conditions associated with an abnormal neurodevelopmental trajectory in infancy and a high prevalence of later NDD in children. As the science of resilience advances, pediatricians should continue to address research programs on the effects of early adverse experiences on well-being and to facilitate a driven enriched environment with early parent-infant interactions.
